# Role of Palliative Radiotherapy in Brain Metastases

**DOI:** 10.4103/0973-1075.53588

**Published:** 2009

**Authors:** Ramesh S Bilimagga, S Nirmala, Karthik S Rishi, MG Janaki, Arul Ponni, AG Rajeev, Suman Kalyan

**Affiliations:** Department of Radiotherapy, M. S. Ramaiah Medical College, Bangalore - 560 054, India; 1 Department of Radiotherapy, Manipal Hospital, Old Airport Road, Bangalore, India

**Keywords:** Brain metastases, Palliative radiotherapy, Whole brain radiotherapy

## Abstract

**Background:**

Brain metastases are a common manifestation of systemic cancer and exceed primary brain tumors in number and are a significant cause of neurologic problems. They affect 20-40% of all cancer patients. Aggressive management of brain metastases is effective in both symptom palliation and prolonging the life. Radiotherapy has a major role to play in the management of brain metastases.

**AIM:**

The aim of the study was to know the outcome of palliative radiotherapy in symptomatic brain metastases in terms of improvement in their performance status.

**Materials and Methods:**

This is a retrospective study of 63 patients diagnosed to have brain metastases and treated with palliative whole brain radiotherapy to a dose of 30 Gy in 10 fractions over two weeks between June 1998 and June 2007. Diagnosis was done in most of the cases with computed tomography scan and in a few with magnetic resonance imaging. Improvement in presenting symptoms has been assessed in terms of improvement in their performance status by using the ECOG scale.

**Results:**

Fifty-four patients completed the planned treatment. Eight patients received concurrent Temozolamide; 88% of patients had symptom relief at one month follow-up; 39/54 patients had a follow-up of just one to three months. Hence survival could not be assessed in this study.

**Conclusion:**

External beam radiotherapy in the dose of 30 Gy over two weeks achieved good palliation in terms improvement in their performance status in 88% of patients. Addition of concurrent and adjuvant Timozolamide may improve the results.

## INTRODUCTION

Brain metastases are the most common intracranial tumors in adults. They affect 20-40% of all cancer patients and represent one of the most frequent neurological complications of systemic cancer as a major cause of morbidity and mortality.[1] The incidence has increased with time probably as a result of advances in treatment of primary tumor and systemic disease which has led to improved survival as well as advances in neuroimaging which has led to early detection of brain metastases. Patients with lung or breast cancer are at greatest risk. The aim of treatment is to improve or maintain quality of life. The various treatment options available are steroids, radiotherapy, surgery, stereotactic radiosurgery, chemotherapy and supportive management. Median survival is one month without treatment, two months with steroids, and three to six months with cranial irradiation.[2]

The present study is a retrospective analysis of all patients of metastatic brain tumor treated with palliative whole brain radiotherapy. The outcome of treatment in terms of symptom relief measured as improvement in performance status and the shortcomings experienced by us in a tertiary care hospital are discussed in this article.

## MATERIALS AND METHODS

This is a retrospective study of all patients diagnosed to have brain metastases and treated with palliative whole brain radiotherapy between June 1998 and June 2007 at a tertiary care hospital. Altogether there were 63 patients. In the majority of patients diagnosis was made with computed tomography (CT) scan and in a few patients with magnetic resonance imaging (MRI). Treatment intent was palliative. Eastern Cooperative Oncology Group (ECOG) scale was used to know the performance status of all patients. All patients were treated with whole brain radiotherapy (RT) to a dose of 30 Gy in 10 fractions over two weeks on Telecobalt machine. Timozolomide was given concurrently with RT at the dose of 100 mg daily in eight cases. Follow-up was advised monthly up to six months and once in three months later. At one month after completion of radiotherapy, response to treatment was assessed in terms of improvement in the performance status. SPSS software was used for statistical analysis. Wilcoxon Signed Rank Test was used to analyze the pre and post-RT performance status. Retrospective analysis of 63 patients diagnosed and treated with RT for brain metastases has been done in the present study.

## RESULTS

Of the 63 cases, male and female patients were almost equal in number (32:31). Age ranged from 34 years to 75 years (median age 56 years). Among males the age ranged from 41-75 years (median 60 years) and among females 34-66 years (range 52 years). All patients presented with some symptoms to the radiotherapy department. The presenting symptoms were headache in 55.5% (35/63) patients, seizures in 20.6% (13/63) patients, 41% (26/63) presented with focal weakness and 25% (16/63) had gait instability and vomiting. Most of the patients had more than one presenting symptom. ECOG performance status was used. Nine patients were in ECOG 1, 20 patients were in ECOG 2, 26 patients were in ECOG 3 and eight patients were in ECOG 4. Fifty-three cases had diagnosed primary. Among them six patients had uncontrolled primary at the time of brain metastases. In ten patients primary was unknown. The diagnosis was made with either CT or MRI. Sixty-eight per cent had supratentorial lesion and 18% had infratentorial lesion and 14% had both the compartments involved. Nearly 86% had multiple lesions. Sixty-four per cent of the lesions were from lung and breast primary (lung 38% and breast 25.5%) [Table 1]. Adenocarcinoma constituted the major histology with 54%, next being squamous cell carcinoma with 22% [Table 2]. Time to diagnose secondary brain lesion from primary varied from 1 to 24 months with a median time of 5.5 months. Fourteen patients had metastases in other organs like lung, liver and bone. Forty-three patients were in recursive partitioning analysis (RPA) Class I, 19 patients in Class II and one patient in Class III at the time of starting radiotherapy.

**Table 1:** Primary Site

Primary	No.
Lung	24
Unknown primary	10
Breast	16
Prostate	02
Colorectum	02
Esophagus	02
Thyroid	02
Melanoma	02
Thymus	01
Stomach	01
Endometrium	01
Total	63

**Table 2:** Histology

Adenocarcinoma	34
Squamous	14
Mucoepidermoid	02
Small cell lung cancer	02
Melanoma	02
Papillary thyroid	02
Lymphoepithelioma	01
Unknown	06
Total	63

Fifty-four patients completed the planned treatment and in nine patients it was incomplete. Among these nine patients, five patients were in ECOG 4 and four patients were in ECOG 3. One patient received re-irradiation for recurrent brain metastasis in a different site by localized fields after a gap of one year. All the patients were followed up. The maximum follow-up was for a duration of 12 months and minimum of one month [Table 3]; 48/54 (88%) patients had improvement in their presenting signs and symptoms at one month follow-up. This was measured in terms of improvement in the performance status. Wilcoxon signed rank test was used to analyze the pre-RT and post-RT values [Table 4]. The *P* value was very significant i.e., less than 0.001. In six patients disease progressed during the follow-up.

**Table 3:** Follow-up

1-3 months	39 cases (5)*
3-6 months	7 cases (2)*
6-12 months	8 cases (1)*

* Timozolamide was given in eight cases

**Table 4:** PRERT* POSTRT Cross Tabulation

	POSTRT					Total
		
	0	1	2	3	4	
PRERT						
Count	5	3	1	-	-	9
% within PRERT	55.6	3.3	11.1	-	-	100.0
Count	1	14	3	2	-	20
% within PRERT	5.0	70.0	5.0	10.0	-	100.0
Count	-	4	18	-	-	22
% within PRERT	-	8.2	81.8	-	-	100.0
Count	-	-	-	1	2	3
% within PRERT	-	-	-	33.3	66.7	100.0
Total						
Count	6	21	22	3	2	54
% within PRERT	11.1	38.9	40.7	5.6	37	100.0

## DISCUSSION

Parenchymal brain metastases are a common manifestation of systemic cancer far outnumbering primary brain tumors and are a significant cause of neurologic problems. They affect 20-40% of all cancer patients, and represent one of the most frequent neurological complications of systemic cancer as a major cause of morbidity and mortality.[1] Certain primary cancers have a predilection for spread to Central Nervous System (CNS). Lung and breast make up about 60% of all brain metastases and metastases from unknown primary 1-18%.[3] In the present study 38% cases were from lung primary and 25.5% cases from breast primary making a total of 64% and from unknown primary 15.4%. Fifty-four per cent of metastases were from adenocarcinoma and 22% from squamous cell carcinoma.

The median interval from diagnosis of cancer to that of brain metastasis is 12 months. In this study it ranged from 1 to 24 months with a median of 5.5 months. Eighty-five per cent of brain metastases are found in the cerebral hemispheres, 10 to 15% are in the cerebellum, and 1 to 3% in the brainstem.[4] In the present study we had 67% tumors in the supratentorium and 17% in the infratentorium and 15.6% were in both the hemispheres. Eighty-five per cent had multiple metastases and 15% had solitary brain metastasis.

The presenting symptoms include headache (49%), focal weakness (30%), mental disturbances (32%), gait ataxia (21%), seizures (18%), speech difficulties (12%), visual disturbance (6%), sensory disturbance (6%), and limb ataxia (6%).[4,5] Fifty-five per cent of our patients had headache and 20% had seizures and 40% presented with focal weakness. Eighty-eight per cent of patients had improvement in their presenting symptoms after completion of palliative radiotherapy at one month follow-up.

The diagnosis is best established by MRI or, alternatively, CT in patients unable to have an MRI scan (e.g., patients with an implanted pacemaker or non-availability). The identification on neuroimaging of an enhancing lesion, commonly at the gray-white matter junction with surrounding edema, in a patient with known cancer usually suffices to establish the diagnosis of brain me tastasis.[2] CT scan was the major imaging modality used to diagnose brain metastases except a few MRI studies in our study. All the patients with unknown primary were diagnosed with the help of CT scan only.

The development of brain metastases is often viewed as the end stage of the disease course. Aggressive management of brain metastases is effective in both symptom palliation and the prolongation of life. The majority of patients with controlled intracranial metastases will expire from systemic disease rather than from recurrence of these metastases.

Surgical excision should be considered for patients with good performance status, minimal or no evidence of extracranial disease, and a surgically accessible single brain metastasis amenable to complete excision. To reduce the risk of tumor recurrence for patients who have undergone resection of a single brain metastasis, postoperative whole brain radiotherapy (WBRT) should be considered. The optimal dose and fractionation schedule for WBRT is 3000 cGy in 10 fractions or 2000 cGy in five fractions.[6] Comparison of surgical resection plus WBRT with surgical resection alone reported possible reduction in the proportion of deaths due to neurological cause as well as less frequency in tumor recurrence at treated site and anywhere in the brain but no significant difference in overall survival or length of functional independence.[6] Surgical excision with WBRT compared to WBRT alone in single brain metastasis is reported to improve functionally independent survival, reduction in death from neurological causes but no improvement in overall survival.[7]

The Radiation Therapy Oncology Group (RTOG) RPA describes three prognostic classes, defined by age, Karnofsky Performance Score (KPS), and disease status.[8] Accordingly the median survival in Class I is 7.1.months and Class II 4.2 months and Class III is 2.3 months. In the present study though 68% (44/63) patients were in Class I RPA and 30%(19/63) in Class II, no survival analysis could be made as no follow-up was found beyond three months in as high as 72% of patients who completed the treatment.

Stereotactic radiosurgery alone appears to be as effective as resection plus WBRT in the treatment of one or two brain metastases for patients in RPA Class 1 and 2.[9] Based on Level I-III evidence, for selected patients with small (up to 4 cm) brain metastases, the addition of radiosurgery boost to WBRT improves brain control as compared to WBRT alone.[10] WBRT and stereotactic boost treatment improved functional autonomy (KPS) for all patients and survival for patients with a single unresectable brain metastasis. WBRT and stereotactic radiosurgery should, therefore, be standard treatment for patients with a single unresectable brain metastasis and considered for patients with two or three brain metastases.[11] After surgical resection of one to two brain metastases, a boost of 10 to 15 Gy in addition to WBRT was found to improve outcome in RPA Class 1 and 2 patients.[12]

Most patients will not be suitable for surgery because of multiple lesions, an inaccessible lesion, active primary disease, or co-morbidity.[6] They are the candidates for palliative WBRT. WBRT has long been the mainstay of definitive treatment, and patients with brain metastases achieve relief of neurologic symptoms for a time with WBRT.[2]

In the present study 88% of patients achieved improvement in their presenting symptoms and signs with WBRT at one month after completion of treatment. The statistical analysis with Wilcoxon signed rank test showed the *P* value to be very significant i.e. less than 0.001. All the patients received WBRT of 30 Gy in 10 fractions. None of the randomized controlled trials with altered dose-fractionation schemes as compared to standard delivery (3000 cGy in 10 fractions) found a benefit in terms of overall survival, neurologic function, or symptom control.[4,6] Dose escalation beyond 30 Gy in 10 fractions does not appear to improve survival or local control in patients with multiple brain metastases but does increase the treatment time and cost of therapy.[13] Short-course WBRT with five fractions of 4 Gy each resulted in survival and local control that were similar to longer programs in breast cancer patients with brain metastases. The dose of five fractions of 4 Gy each appears preferable to the majority of these patients because it is less time-consuming and more convenient.[14] Higher cumulative doses (>50 Gy), daily fractions >2 Gy, concurrent chemotherapy, and patient age >60 years are all factors that increase the rate of complications, such as acute and delayed encephalopathies and radiation necrosis.[2,5]

Timozolomide has been tried concurrently with RT in metastatic brain tumor also and has shown good radiological response and reduces the need of corticosteriods.[1] Results of a randomized Phase II trial of concurrent administration of temozolomide and radiation therapy followed by adjuvant temozolomide therapy compared with radiation alone showed a higher rate of complete and partial responses (objective response of 96% vs. 67%) and significantly more complete responses (38% vs. 33%, *P* =.017), primarily in patients with newly diagnosed brain metastases. There was marked neurologic improvement as well as less corticosteroid dependency two months after treatment in the group receiving temozolomide(67% vs. 91%).[15,16] Due to cost factor, only eight of our patients received concurrent Timozolomide, but no one completed adjuvant cycles.

Other chemotherapeutic agents that have shown promising results in metastatic brain tumors are Topotecan,[17] and Capecetabine, particularly in metastasis from breast cancer.[18] The addition of efaproxiral, a non-cytotoxic radiation sensitizer to WBRT may improve results in patients with brain metastases from breast cancer.[19,20] Phase III trials to assess the benefit of motexafin in patients with metastatic lung cancer and efaproxiral in patients with metastatic breast cancer are ongoing.[21]

### Shortcomings in the present study

Though we had a good number of patients (63), follow-up is poor (72% less than three months follow-up) in spite of repeated reminders to the patients. Though 44 patients were in RPA Class I no correlation could be made with regard to treatment outcome due to poor follow-up. Due to cost factor only eight patients received timozolamide and only in two cases the follow-up extended beyond four months hence benefits could not be assessed.

## CONCLUSIONS

External beam radiotherapy in the dose of 30 Gy over two weeks achieved good palliation in terms of symptom improvement in 88% of patients. Addition of concurrent and adjuvant Timozolamide may improve the results. Short-course radiotherapy in the form of 4 Gy per fraction × five sittings can be practiced, which will reduce the cost of treatment as well as patients’ hospital stay.
